# Quality of Reporting on the Vegetative State in Italian Newspapers. The Case of Eluana Englaro

**DOI:** 10.1371/journal.pone.0018706

**Published:** 2011-04-12

**Authors:** Nicola Latronico, Ottavia Manenti, Luca Baini, Frank A. Rasulo

**Affiliations:** Department of Neuroanesthesia and Neurointensive Care, University of Brescia, Spedali Civili, Brescia, Italy; Dalhousie University, Canada

## Abstract

**Background:**

Media coverage of the vegetative state (VS) includes refutations of the VS diagnosis and describes behaviors inconsistent with VS. We used a quality score to assess the reporting in articles describing the medical characteristics of VS in Italian newspapers.

**Methodology/Principal Findings:**

Our search covered a 7-month period from July 1, 2008, to February 28, 2009, using the online searchable databases of four major Italian newspapers: Corriere della Sera, La Repubblica, La Stampa, and Avvenire. Medical reporting was judged as complete if three core VS characteristics were described: patient unawareness of self and the environment, preserved wakefulness (eyes open), and spontaneous respiration (artificial ventilator not needed). We retrieved 2,099 articles, and 967 were dedicated to VS. Of these, 853 (88.2%) were non-medical and mainly focused on describing the political, legal, and ethical aspects of VS. Of the 114 (11.8%) medical articles, 53 (5.5%) discussed other medical problems such as death by dehydration, artificial nutrition, neuroimaging, brain death, or uterine hemorrhage, and 61 (6.3%) described VS. Of these 61, only 18 (1.9%) reported all three CORE characteristics and were judged complete. We found no differences among the four investigated newspapers (Fisher's exact  = 0.798), and incomplete articles were equally distributed between journalistic pieces and expert opinions (χ^2^ = 1.8854, P = 0.170). Incorrect descriptions of VS were significantly more common among incomplete articles (13 of 43 vs. 1 of 18; Fisher's exact P = 0.047).

**Conclusions/Significance:**

Core VS characteristics are rarely reported in Italian newspaper articles, which can alter adequate comprehension of new developments and (mis)inform political, legal, and ethical decisions.

## Introduction

The popular media are commonly the initial source of medical research news for both medical professionals and the public, and interest in reporting quality is widespread[1], [2].

Studies of the accuracy and completeness of newspaper reporting of scientific issues have found that most newspaper articles contain errors or omit important information and include little scientific explanation or critique of the quality or relevance of scientific evidence[3], [4], [5], [6], [7], [8].

Vegetative state (VS) is a clinical condition of wakefulness without awareness caused by traumatic and non-traumatic brain injuries[9], [10]. Racine et al. recently reviewed how American print media depicted the neurologic condition, behavioral repertoire, prognosis, and withdrawal of life support of Terri Schiavo, a patient with persistent VS after cardiac arrest. Quality of reporting was not explicitly evaluated; however, these authors found that a significant proportion of news media articles described behaviors inconsistent with VS[11]. The Racine's study also demonstrated a general absence of adequate background information about VS, which is an essential pre-requisite for informed political, legal, and ethical decisions. As noted in an accompanying Editorial[12], a videotape showing that Ms. Schiavo was able to open and move her eyes ignited the public skepticism over her diagnosis of VS because most viewers had no idea that VS patients can have periods of sleep alternating with periods of an awake-like state in which eyes are open and may move about.

Standardized methods for assessing the quality of reporting may help improve the reliability of such articles, and can be worthwhile in pointing out areas of strength and deficiencies and in suggesting strategies for improvement. We used a quality score specifically developed for the study to examine print media coverage of the case of Eluana Englaro, a young Italian patient who died after 18 years in a VS, to investigate the quality of reporting in Italian newspaper articles describing the medical characteristics of VS.

### Case background

Eluana Englaro was 39 years old when she died after 18 years in a VS after a car accident in January 18, 1992. Death occurred soon after the withdrawal of artificial hydration and nutrition (AHN); however, withdrawal took place months after the Italian Supreme Court had ruled, for the first time in Italy, that AHN should be stopped and Eluana Englaro allowed to die. During this interim period, all of the circumstances surrounding Eluana Englaro's life and death became a matter of highly polarized public discourse, and the decision to withdraw AHN became the crossroads at which law, politics, religion, and medicine collided, attracting attention at the highest levels of political power[13], [14], [15], [16], [17].

According to her father Beppino Englaro and her friends, Eluana had expressed prior wishes that she did not want futile medical care[15], [16]. Mr. Englaro proposed removing the feeding tube of her daughter since 1996 [13], starting a long legal battle, which is synthesized in the following text (for review, see [15], [18]).

The case was debated in court starting in December 1999 when the request of Englaro's father to have her feeding tube removed was rejected by the Milan Court of Appeal. In the following years the request was repeatedly refused: by the Milan Court of Appeal in July 2002, by the Supreme Court of Cassation in April 2005, by the First Instance Court in February 2006, and again by the Milan Court of Appeal in December 2006. Motivations for dismissing the appeal varied. First Instance Court's decision was based on the fact that refusal of medical treatment falls into the category of “strictly personal legal acts”, which cannot be performed by the “guardian” [18]. The Milan Court of Appeal dismissed the appeal on the basis that Eluana had expressed her desire not to be kept alive artificially when experiencing profound emotional distress after she had seen a friend in an irreversible coma[18]. In addition, the Court asserted that life is a supreme good, which prevails over the right to refuse treatment[18].

On October 17, 2007, the Supreme Court of Cassation overturned the Milan Court of Appeal's decision and granted a request for a new trial by contemplating the possibility for a person to “refuse the therapy or to knowingly decide to interrupt it, in all phases of one's life, even in the terminal phase”, provided that VS is irreversible and there is no evidence of even minimal recovery of consciousness and perception of the external world, and the request is consistent with the patient's will[18]. The Supreme Court also distinguished treatment refusal (‘the expression of the sick person’s intention to choose that the disease follows its natural course') from euthanasia (‘a behavior aiming at shortening an individual’s life, actively causing death’) stressing out that the doctor's ethical and legal obligation to treat is based on the patient's consent, in the absence of which the obligation ceases[18]. On July 9, 2008, the Milan Court of Appeal issued an order to suspend AHN. The Italian Parliament brought a jurisdictional conflict before the Final Court of Appeal, asserting that the decision was changing existing laws, but the Court rejected this request on October 10, 2008. On November 13, 2008, Italy's highest court awarded Englaro's father the right to stop his daughter's AHN. On December 16, 2008, the Minister of Health issued a ‘Nota’ (a sub-legislative act) forbidding the interruption of treatment for disabled people hospitalized within the National Health Service. The act was interpreted by the Radical Party as intimidating for Eluana's leading to a criminal investigation against the Minister. On January 6, 2009 the Italian prime minister's cabinet issued a decree impeding doctors from withdrawing AHN, effectively overruling the country's top judges; however, the president of the Republic refused to sign the decree, which was required for it to become law. On January 7, 2009 a ministerial inspection found some “administrative anomalies” regarding the use of the hospital room where Eluana had been admitted.

On February 2, 2009 Eluana was transferred to La Quiete, a private nursing home in Udine, where the withdrawal of ANH. On February 6, 2009, the withdrawal protocol started. Eluana Englaro died on February 9, 2009, at 19:35, while the Italian Senate was holding a turbulent emergency session to pass a bill forcing doctors to resume AHN[15], [16]. On February 27, 2009 Mr. Englaro was charged with murder, along with the chief anesthesiologist Dr. Amato De Monte and the 12 people of his staff that assisted Eluana at the end of her life. On January 11, 2010 Englaro, De Monte and all members of the medical staff were cleared of murder charge[19].

## Methods

### Article search strategy

We searched the online databases of four major newspapers in Italy during a 7-month period from July 1, 2008 (the Court of Appeal ruled that AHN should be stopped) to February 28, 2009 (the death of Eluana). We selected the three newspapers with the highest circulation in the country, Corriere della Sera, La Repubblica, and La Stampa, and Italy's major Catholic newspaper, Avvenire.

To maximize search yields, we searched the words “vegetativo”, “Eluana,” or “Englaro” as mutually exclusive terms on headlines, lead paragraphs, and body text. Two medical students (O.M. and L.B.) read the articles independently and classified them as political, legal, ethical, personal, religious, social, or medical.

### Quality score

To evaluate the quality of the reporting in newspapers articles describing the medical characteristics of VS, we devised a scoring system designating them as complete or incomplete. The following steps were taken to develop the scoring system[20], [21]: 1) item generation; 2) pretesting with item aggregation; 3) inter-rater and test-retest reliability; and 4) construct validity. The literature review and expert opinion resulted in the generation of three items defined as essential for a proper medical description of VS, which were designated as CORE (**C**onsciousness, eyes **O**pen, spontaneous **RE**spiration) characteristics: unawareness, wakefulness, and spontaneous respiration. Unawareness and wakefulness are universally reported as distinguishing features of the VS [9], [22], [23]. Compared to other autonomic functions, spontaneous respiration is a further essential descriptor[9], [24] that gives immediate evidence of the capacity of VS patients to survive with ordinary means outside intensive care units, although the contingent use of a ventilator is possible for some patients with spinal cord injury or acute respiratory distress.

After a pilot study of 30 articles that two raters (O.M., L.B.) scored independently and that the study group subsequently discussed in detail, it was decided that only articles reporting all three CORE characteristics of VS should be classified as complete. Each of the CORE characteristics can be observed in other chronic disturbances of consciousness, but it is only in VS that all three are present[25].

Inter-rater reliability was tested on the 61 articles describing the medical characteristics of VS. Two raters (O.M., L.B.) reviewed all articles independently and blinded to authorship and origin.

Test–retest reliability for quality reporting was determined through the duplicate scoring of 30 articles by the same rater (O.M.) with an interval of 8 weeks.

To examine construct validity, we hypothesized that incorrect descriptions of VS would be more frequent in articles judged to be incomplete than in articles judged to be complete.

Articles judged as complete were further evaluated for completeness of reporting. We evaluated if articles described the VS patient as completely depending on others for the activities of daily life; requiring nursing assistance because of fecal and urinary incontinence and mobilization and other measures to prevent pressure ulcers; having muscle atrophy because of being bedridden; and needing AHN, usually through a naso-gastric tube because of an inability to feed independently. Articles rated as complete were also evaluated for reporting on the issue of internal consciousness, i.e., addressing the evidence by means of neurophysiologic and functional neuroimaging studies that patients clinically diagnosed with VS may indeed have residual cognitive abilities.

### Data presentation and statistical analysis

We used descriptive statistics to characterize the composition and properties of the sample, and assessed inter-rater variability concerning the classification of topics (political, legal, ethical, personal, religious, medical, and social) and reporting quality.

Inter-rater agreement was “nearly perfect”[26] for both the quality of reporting scores (k = 0.96; standard error  = 0.128) and classification of topics (k = 0.95; standard error  = 0.011). Test–retest reliability was good with identical scores for the first and second evaluations (k = 1.00; standard error  = 0.183).

Differences in the proportions of incomplete articles among different newspapers, of incomplete articles between journalistic articles and expert opinions, and of incorrect descriptions of VS between complete and incomplete articles were analyzed in contingency tables by means of the χ^2^ statistics, or the Fisher's exact test when the expected values in any of the cells of the contingency table was less than 5. We used STATA, version 10 (StataCorp, College Station, Texas), for all analyses.

## Results

**Figure 1:**
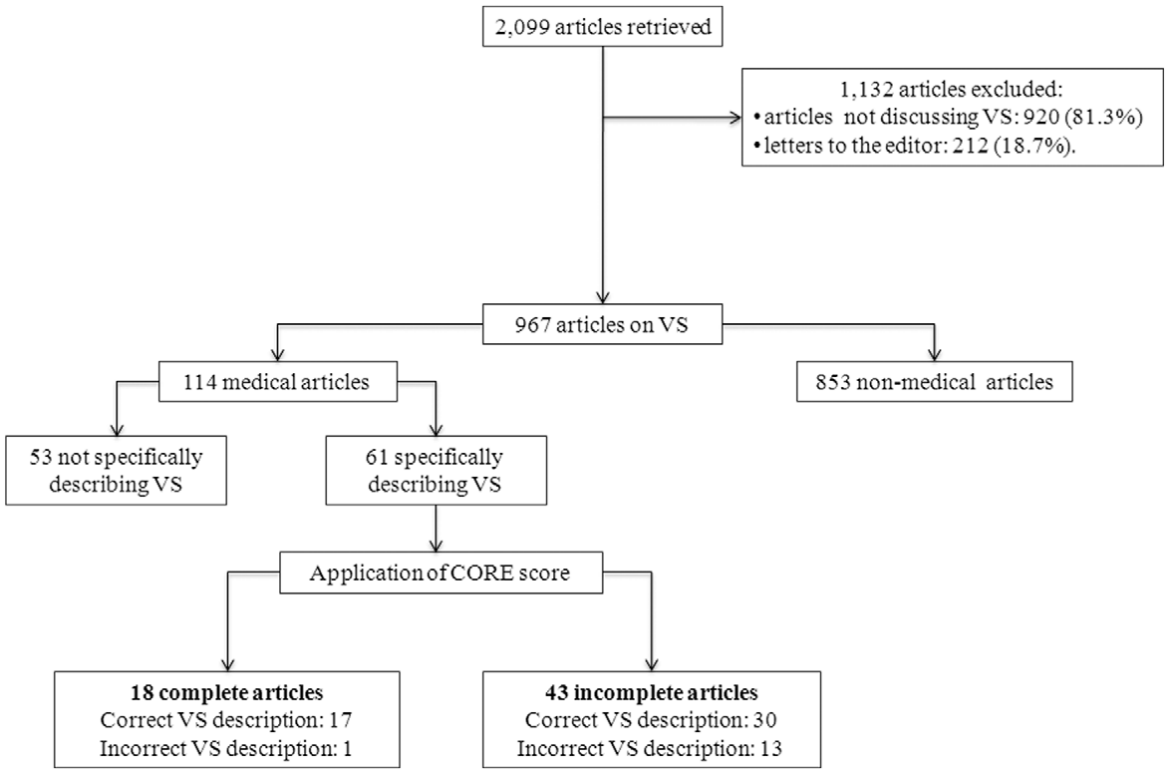
Flow chart of the study protocol. According to the CORE score (**C**onsciousness, eyes **O**pen, spontaneous **RE**spiration), the articles were judged as complete if they described all three major characteristics of VS: unawareness, wakefulness and spontaneous respiration. VS indicates vegetative state.

We retrieved a total of 2,099 articles published in the Avvenire (n = 837; 39.9%), La Repubblica (n = 491; 23.4%), La Stampa (n = 454; 21.6%), and Corriere della Sera (n = 317; 15.1%). A total of 1132 publications were excluded because they did not discuss VS (n = 920, 81.3%) or were letters to the editor (n = 212, 18.7%) (Fig. 1).

Of the remaining 967, 853 (88.2%) were non-medical articles, and 114 (11.8%) were medical articles. The analysis of the 967 articles generated 1651 topics (Table 1). Political, legal, and ethical topics dominated in La Repubblica, La Stampa, and Corriere della Sera, whereas legal, ethical, and personal pieces dominated in Avvenire.

**Table 1:** Topics in 967 print media articles of the Englaro case.

NEWSPAPERS										
TOPICS	*La Repubblica*		*La Stampa*		*Corriere della Sera*		*Avvenire*			
	No of topics	Percentage	No of topics	Percentage	No of topics	Percentage	No of topics	Percentage	Total number of topics	Percentage
NON MEDICAL										
political	168	29.3	119	28.1	94	26.4	37	12.4	418	25.3
legal	133	24.4	87	20.6	81	22.8	88	29.4	389	23.6
ethical	108	19.8	95	22.5	63	17.7	67	22.4	333	20.2
personal	78	14.3	49	11.6	56	15.7	45	15.1	228	13.8
religious	54	9.9	45	10.6	27	7.6	18	6.0	144	8.7
social	5	0.9	6	1.4	7	2.0	7	2.3	25	1.5
MEDICAL	27	4.9	22	5.5	28	7.9	37	12.4	114	6.9
Tot.	573		423		356		299		1651	100.0

The total number of topics (n. 1651) is greater than the total number of articles (n. 967) because one article could be classified in more than one topic. Percentages refer to the total number of topics.

Of the 114 medical articles, 61 (6.3% of 967) described the condition of VS, whereas 53 discussed other medical problems such as the pathophysiology of death by dehydration; the technical requirements for, and the goals and the complications of the artificial nutrition; head trauma as a major cause of the VS; the definition and diagnosis of brain death (Table 2). Uterine hemorrhage complicated the clinical course of Eluana Englaro's during the investigated period and was discussed in 10 articles.

**Table 2:** Medical media articles.

NEWSPAPERS					
	*La Repubblica*328 articles	*La Stampa*251 articles	*Corriere della Sera*209 articles	*Avvenire*179 articles	*Total number of headline topics*
Articles specifically describing the vegetative state	8	11	19	23	61
Articles not specifically describing the vegetative state	19	11	9	14	53
Death by dehydration (pathophysiology)	4	3	6	2	15
Uterine hemorrhage	3	2	1	4	10
Artificial nutrition (technical aspects)	3	3	0	3	9
Head trauma (neuroimaging)	3	1	2	2	8
Brain death	5	0	0	0	5
Other medical aspects	1	2	0	3	6
Tot.	27	22	28	37	114

Of the 61 articles describing VS, 29 were news reports or features and 32 reported expert opinions. Eighteen of these 61 articles (29.5%) reported all three CORE characteristics and were judged complete. Further, all of these articles also described the general condition of complete dependency of the VS patient, and none discussed the problem of residual cognitive abilities in patients clinically diagnosed with VS (Table 3).

**Table 3:** Quality of reporting of articles specifically describing the vegetative state.

NEWSPAPERS					
QUALITY OF REPORTING	*La Repubblica*27 articles	*La Stampa*22 articles	*Corriere della Sera*28 articles	*Avvenire*37 articles	Tot.
Incomplete	7	8	13	15	43
Complete* description of complete dependency* * description of residual cognition*	110	330	660	880	180
Tot.	8	11	19	23	61

Only 18 articles (1.9% of 967 articles) reported a complete description of the vegetative state according to the CORE (**C**onsciousness, eyes **O**pen, spontaneous **RE**spiration) score.

Forty-three of 61 articles (70.5%) were judged to be incomplete because they did not report one or more of the 3 CORE characteristics of VS (Table 4), with no differences among the newspapers (Fisher's exact  = 0.798). Incomplete articles were less common among journalistic articles than among those reporting expert opinion; however, the difference was not statistically significant (18 of 29 [62.1%] vs. 25 of 32 [78.1%], respectively; χ^2^ = 1.8854, P = 0.170). Unawareness was the most commonly described CORE characteristic and spontaneous respiration the least described (Table 4). In nine articles, none of the three CORE characteristics was reported; instead, other aspects of VS were described, such as artificial nutrition via naso-gastric tube, (in)ability to swallow, or a description of recent achievements with functional neuroimaging with no attempt to put this information into the context of wakeful unawareness in VS patients.

**Table 4:** Reporting of the CORE score components in the 61 articles specifically describing the vegetative state.

NEWSPAPERS					
	*La Repubblica*	*La Stampa*	*Corriere della Sera*	*Avvenire*	*Tot.*
All 3 CORE score components reported (unawareness, wakefulness, spontaneous respiration)	1	3	6	8	18
Only 2 CORE score components reportedwakefulness + spontaneous respirationunawareness + wakefulnessunawareness + spontaneous respiration	3120	3102	2011	4310	12543
Only 1 CORE score component reportedunawarenesswakefulnessspontaneous respiration	3210	4310	5401	10712	221633
None reported	1	1	6	1	9
Tot.	8	11	19	23	61

According to the CORE score (**C**onsciousness, eyes **O**pen, spontaneous **RE**spiration), the articles were judged as complete if they described all three major characteristics of VS: unawareness, wakefulness and spontaneous respiration.

Incorrect VS descriptions were more frequent in articles reporting expert opinions than in journalistic articles; however, the difference was not statistically significant (4 of 29 [13.8%] vs. 10 of 32 [31.2%]; Fisher's exact P = 0.134).

## Discussion

Our evaluation of Italian print media coverage of the Eluana Englaro case identified substantial shortcomings in journalistic reporting. The majority of articles (88%) were dedicated to nonmedical aspects of VS. A minority (12%) addressed medical issues. Articles describing the medical characteristics of VS were even less frequent (6.3%), and their quality of reporting was rarely complete. Inadequate quality of reporting was mostly the result of missing information, although incorrect VS description was also present. Incorrect descriptions were twice as frequent in articles reporting expert opinions as in journalistic articles, although this difference was not statistically significant. This suggests that experts, whose duty should be “to clarify, explain, and caution”[27], did not effectively contribute to clarify nor to explain the complex issue of VS.

These results are similar to those obtained by Racine et al. in a US study of media coverage of VS[11]. They examined 1,141 articles published on the case of Terri Schiavo in four North American newspapers. Schiavo had remained in a VS for 15 years before dying after enteral nutrition was stopped in 2005, and the most frequent headline themes featured the legal (31%), end-of-life (25%), and political (22%) aspects of the case. The medical and scientific aspects of the case, such as information about diagnosis and treatment, were found in 9% of headlines[11]. Quality of reporting was not explicitly evaluated; however, only 1.4% of articles provided an explanation of VS, with a high number of incorrect or equivocal descriptions[11].

We can only speculate on the reasons why the medical aspects of VS were rarely considered to be newsworthy. Newspapers have limited space, and articles about medicine compete with other stories [1], [3], [29]. The decision to publish reflects established news values such as timeliness, novelty, impact, proximity, conflict, prominence, and human interest. Stories that are not considered newsworthy are not printed, and medically worthy information is not necessarily newsworthy[1]. In a recent analysis, only 0.7% of available printed space was given to health-related issues in Italian newspapers[30].

Some journalists will say that “their paramount role is to report the news” [31]. However, strict adherence to what is considered newsworthy can be deleterious for proper coverage of complex health-care–related topics[32]. As Dentzer recently noted[31], when journalists ignore complexities or fail to provide context, the public health messages they convey are inevitably inadequate or distorted. Absence of novelty could have been a reason not to consider a VS description newsworthy. The original VS definition dates back to 1972[9]; the term “vegetative” might itself be considered to be intuitive enough to make explanation useless. Jennett and Plum justified the need for defining VS to “accurately understand and discuss” situations “never previously encountered”, and cited the Oxford English Dictionary to explain their choice of the term vegetative: “to vegetate is to live a merely physical life devoid of intellectual activity or social intercourse,” and vegetative describes “an organic body capable of growth and development but devoid of sensation and thought” [9].

There are, however, several reasons to consider VS as a complex, evolving, and controversial medical issue[33], [34], [35], that is thus newsworthy. First, lay people as well as health-care professionals may not really understand VS or be reluctant to accept that VS can be reliably diagnosed. In a North American survey, 22% of family members of VS patients and 30% of physicians considered the patient to have some sort of awareness of pain and suffering[36]; a recent European survey of 2,059 medical and paramedical professionals showed that a high proportion of medical doctors (56%) and paramedical professionals (68%) considered that VS patients may feel pain[37]. Second, the field of chronic alterations of consciousness substantially changed after the introduction in 2002 of the definition of the minimally conscious state, an altered consciousness in which minimal but definite behavioral evidence of self or environmental awareness is demonstrated[38]. Both VS and a minimally conscious state are syndromes with a clinical diagnosis; however, differential diagnosis is difficult and prone to errors[39]. Third, time limits for permanent VS, usually one year after traumatic brain injury and 3 to 6 months after non-traumatic brain injury[22], [23], have been brought into question by recent follow-up studies showing that VS patients may recover consciousness even after years, although with severe functional disability[40], [41]. Finally, recent studies indicate that some patients clinically diagnosed with VS can in fact retain residual cognitive abilities[42], [43], [44], [45]. Interestingly, recent studies on functional neuroimaging[44] were mostly cited by Italian journalists, implying that they were aware of the limitations of the clinical diagnosis of VS and of the intricacies surrounding the definition of consciousness. We interpret this observation as further evidence of uncertainty about the proper role of the journalist, whether it is to describe the bigger picture or simply to report what is new[31].

### Strengths and limitations of the study

This study is the first to evaluate the completeness of newspapers reporting on VS using a pre-defined scoring system, as in reporting of primary studies in medical journals[28]. The development of the scoring system met current methodological standards[20], [21]. Reliability was excellent with nearly perfect agreement for both inter-observer and test-retest variability. Importantly, we found that articles judged to be incomplete were more likely to report incorrect description of VS, thus confirming the construct validity of the scoring system. We did not formally test sensibility or credibility; however, constitutive items were simple, and were devised after consultation with experts in the field.

This study also has limitations. First, we used the search engines built into each individual newspaper's website. Differences among the search engines could have led to a variability of results among the newspapers. Second, content analysis coding always involves subjectivity. The high level of agreement observed among coders, however, is reassuring. Third, our findings are based on four newspapers, which may raise concern about generalizability; however, the selected newspapers have the highest circulations in Italy, and the results are similar to those obtained in the USA, suggesting a generalized trend or a more general trend. Finally, the CORE score has only been tested on the current dataset; replication of our results using other datasets is needed before its validity can be better ascertained.

### From Quinlan to Englaro: settled issues reopened

VS has frequently engendered significant legal and ethical controversy, particularly about the appropriate degree of life-sustaining treatment. The most important VS case is *In re Quinlan* [46]. In 1975 Karen Ann Quinlan was 21 years old when she suffered a cardiopulmonary arrest after accidental ingestion of a combination of sedatives and alcohol, causing a devastating brain damage and leaving her in a permanent VS. In the first year after cardiac arrest, Karen's biologic functions were maintained by a mechanical ventilator and AHN. Her father sought judicial appointment to be Karen's legal guardian with authority to remove the ventilator. In 1976, the New Jersey Supreme Court's decision upheld the father's petition to remove Karen's ventilator, because she had irretrievably lost the possibility to return to a “cognitive sapient state”. The court ruled that Karen had a constitutional right to refuse medical interventions, that she had not lost her right by becoming incompetent, and that her parents could exercise it on her behalf based on what they thought she would want done[47]. The landmark New Jersey Supreme Court's decision sanctioned the self-determination at life's end in the USA, and the Karen's case became known under the slogan “right to die,” even though the case did involve a right to refuse medical treatment[47]. The public discovered that VS patients breath spontaneously, because Quinlan survived another 9 years in a VS after she was disconnected from the ventilator[48].

The case of Nancy Cruzan got to the United States Supreme Court in 1990[49]. In 1983 Nancy Cruzan, aged 26, suffered a traumatic brain injury and was left in the same condition as Karen Quinlan, except that she needed only a feeding tube to continue to live[50]. Her parents affirmed that she would not want to have tube feeding continued under such circumstances. For this reason, a trial judge authorized her parents to have their daughter's tube feeding removed and AHN discontinued. The Missouri Supreme Court reversed the decision, helding that Cruzan's right to refuse treatment was personal to her, and no one could exercise it on her behalf[49]. The court decided that AHN could be stopped only if it could be shown by "clear and convincing evidence" that Cruzan herself had rejected such treatment. Cruzan's parents appealed this decision to the U.S. Supreme Court. The Supreme Court ruled that the Missouri Supreme Court had the constitutional authority to require clear evidence that the individual personally made the treatment refusal; the Court also affirmed that AHN should be treated like all other medical treatment (especially like ventilators). After the opinion, three of Nancy's friends testified that she had told them that if she did not want to be tube fed, and AHN was stopped. Nancy Cruzan died on December 26, 1990, shortly after her feeding tube was removed.

Despite a number of state courts in the USA echoed *Quinlan* and *Cruzan* on the patient's prerogative to refuse treatment[51], the recent case of Theresa Marie (Terri) Schiavo seemed to call into question past legal and ethical consensuses[52]. In 1990, Theresa Marie (Terri) Schiavo, 26 years of age, had a cardiac arrest and experienced severe hypoxia for several minutes. By late 1990, Mrs. Schiavo was determined to be in a persistent VS. Fifteen year later, on March 18, 2005, after a long dispute between Terri's husband and legal guardian Michael Schiavo and her parents, Robert and Mary Schindler, the Florida Supreme Court ruled that AHN could be removed. Despite the intervention of Congress and President George W. Bush[53], AHN was not resumed and Terri Schiavo died on March 31, 2005.

Having the courts reached a broad consensus on the right to refuse life-sustaining treatment, why did the case of Schiavo cause such a turmoil among the political leaders and the public? Were the causes different in the case of Englaro in Italy? As Annas recently observed[53], “conflicts involving medical decision making for incompetent patients near the end of life are no longer primarily legal in nature, if they ever were”. The collision between two different conceptions of life was the major reason fuelling the debate about the Schiavo and Englaro cases, “a struggle between sanctity of life versus quality of life”[54], with contending parties divided into social conservatives, who believe in the intrinsic value of all life and social liberals, who emphasize quality[55]. A related cause was whether AHN should be considered as a form of medical treatment that can be interrupted if deemed to be futile or rather basic health care that should never be withdrawn. The former position has been endorsed by medical societies[56], [57], and by the 2000 Document of the Italian Ministry of Health Commission on Nutrition and Hydration[58]; more recently, however, this issue has split the Italian National Committee for Bioethics in two opposite views[59]. The latter position has been strongly supported by the Roman Catholic Church, as addressed by Pope John Paul II in 2004[60]: “…the administration of water and food, even when provided by artificial means, always represents a natural means of preserving life, not a medical act. Its use, furthermore, should be considered in principle, ordinary and proportionate, and as such morally obligatory”. A final reason was whether allowing feeding tube to be removed would initiate a slippery slope leading to treatment limitations for disabled persons, or to legalizing assisted suicide or euthanasia[13], [54].

### Conclusions

Commentators have frequently observed that the media fuelled the controversy about VS by providing incorrect representation of VS[11], [13], [54], which is substantiated by our results.

Our results indicate an urgent need to improve the quality of newspaper reporting of the medical aspects of VS. VS is newsworthy, provided that scientific evidence is evaluated rigorously: a clear description of the CORE characteristics of VS may help people put new developments into the appropriate context. VS has strong legal, ethical and political implications, with many questions remaining[61], including issues of diagnosis of VS with the medical and ethical imperative to keep a clear distinction between VS and the minimally conscious state [62], [63], establishing permanence, the decision to withdraw feeding, mode of death, and who best represents the patient's wishes.

These questions will continue to create intense debate. A clear description of the CORE characteristics of VS would be important considering that an accurate explanation of the basic facts necessary to understand the issue is vital for informed political, legal, and ethical decisions.
